# Stroke‐induced excess in capillarization relative to oxidative capacity in rats is muscle specific

**DOI:** 10.14814/phy2.16153

**Published:** 2024-07-17

**Authors:** Hans Degens, Arjun Paudyal, Gert Kwakkel, Mark Slevin, Huub Maas

**Affiliations:** ^1^ Department of Life Sciences Manchester Metropolitan University Manchester UK; ^2^ Institute of Sport Science and Innovations Lithuanian Sports University Kaunas Lithuania; ^3^ Department of Human Movement Sciences, Faculty of Behavioural and Movement Sciences Amsterdam Movement Sciences, Vrije Universiteit Amsterdam Amsterdam The Netherlands; ^4^ Department of Rehabilitation Medicine Amsterdam Movement Sciences, Amsterdam Neuroscience, Amsterdam UMC, Vrije Universiteit Amsterdam Amsterdam The Netherlands; ^5^ Department of Physical Therapy and Human Movement Sciences, Feinberg School of Medicine Northwestern University Chicago Illinois USA; ^6^ Department of Neurorehabilitation Amsterdam Rehabilitation Research Centre Amsterdam The Netherlands; ^7^ The George Emil Palade University of Medicine, Pharmacy, Science, and Technology of Targu Mures Targu Mures Transylvania Romania

**Keywords:** atrophy, muscle morphology, oxygen consumption, photothrombotic stroke, stroke

## Abstract

Stroke is not only associated with muscle weakness, but also associated with reduced muscle fatigue resistance and reduced desaturation during exercise that may be caused by a reduced oxidative capacity and/or microvasculature. Therefore, the objective of the present study was to determine the effects of stroke on muscle mass, fiber size and shape, capillarization and oxidative capacity of the rat *m. extensor carpi radialis* (ECR) and *m. flexor carpi ulnaris* (FCU) after a photothrombotic stroke in the forelimb region of the primary sensorimotor cortex. The main observation of the present study was that 4 weeks after induction of stroke there were no significant changes in muscle fiber size and shape. Although there was no significant capillary rarefaction, there was some evidence for remodeling of the capillary bed as reflected by a reduced heterogeneity of capillary spacing (*p* = 0.006) that may result in improved muscle oxygenation. In the ECR, but not in the FCU, this was accompanied by reduction in muscle fiber oxidative capacity as reflected by reduced optical density of sections stained for succinate dehydrogenase (*p* = 0.013). The reduced oxidative capacity and absence of significant capillary rarefaction resulted in a capillary to fiber ratio per unit of oxidative capacity that was higher after stroke in the ECR (*p* = 0.01), but not in the FCU. This suggests that at least during the early stages, stroke is not necessarily accompanied by muscle fiber atrophy, and that stroke‐induced reductions in oxidative capacity resulting in relative excess of capillarization are muscle specific.

## 1 INTRODUCTION

Stroke is the second global cause of death and the third global cause of disability in both developed and developing nations (Feigin et al., 2017), but as many as 70% of people suffering a stroke survive for at least 5 years (Radisauskas et al., 2019). Most stroke survivors experience motor disabilities (Sunnerhagen et al., 1999) and impaired functional performance (Gianuzzi et al., 2001; Severinsen et al., 2016) that are largely due to muscle weakness consequent to an impaired cortico‐spinal motor control of affected muscles (Qi et al., 2024).

A decreased descending drive is, however, not the only cause of muscle atrophy and weakness after stroke. The weakness and atrophy may be aggravated by lower physical activity levels and undernutrition (Hunnicutt & Gregory, 2017; Ivey et al., 2010; Qi et al., 2024; Ryan et al., 2002), and fiber type grouping and the presence of angular fibers seen in a substantial number of patients (Slager et al., 1985) may reflect denervation and reinnervation following stroke. The loss of force generating capacity has been reported to be larger in plantar flexors than knee extensors, despite a more pronounced atrophy in thigh than lower leg muscles (Hunnicutt & Gregory, 2017), and thigh lean mass was reduced less than arm lean mass (Ryan et al., 2002) suggesting a muscle‐specific response to stroke.

In humans, besides muscle fiber atrophy (Slager et al., 1985), loss of muscle mass (Adkins et al., 2021) and muscle strength, studies also report a stroke‐induced reduction in muscle fatigue resistance (Gerrits et al., 2009; Gianuzzi et al., 2001; Qi et al., 2024). A reduced vasodilatory function (Ivey et al., 2010), and hence a reduced blood flow during muscle contractile activity, may well contribute to the lower muscle fatigue resistance, as also suggested by the association with a reduced peak aerobic fitness (Ivey et al., 2010). The reduced resting, hyperemic (Ivey et al., 2010) and contraction‐induced flow (Murphy et al., 2019) that will reduce shear stress on endothelial cells—needed for the maintenance of the capillary bed (Hudlicka et al., 1992)—may result in capillary rarefaction that has been shown in rats to result in reduced muscle fatigue resistance independent of changes in blood flow, oxidative capacity or fiber type composition (Tickle et al., 2020). The lower hemoglobin desaturation—reflecting a lower oxygen extraction—in the paretic than the non‐paretic leg during exercise (Hyngstrom et al., 2023) may therefore be a consequence of capillary rarefaction and/or a reduced aerobic capacity of the muscle fibers, but this has hitherto not been explored. Other factors that may contribute to an increased muscle fatigability are type I–type II fiber transition and reduction in muscle oxidative capacity (Severinsen et al., 2016). Much of the above‐mentioned stroke‐induced muscle dysfunction found in humans may thus be attributable to changes in muscle fiber type composition, size, metabolism and/or capillarization.

Like in stroke patients, rodent models of ischemic stroke suffer from a reduction in “skilled reach performance” (van Lieshout et al., 2021), and muscle atrophy and weakness (Choe et al., 2006; Springer et al., 2014; Tuntevski et al., 2020) that were at least partly explicable by concomitant muscle fiber atrophy (Choe et al., 2006). Therefore, the aim of the present study was to determine in rats: (i) the effects of stroke on muscle mass, fiber size and shape, capillarization and oxidative capacity, and (ii) whether the changes differed between the *m. extensor carpi radialis* (ECR) and *m. flexor carpi ulnaris* (FCU) 4 weeks after a photothrombotic stroke.

## 2 MATERIALS AND METHODS

### 2.1 Animals

Young‐adult male Sprague–Dawley rats were divided into two groups: 4 weeks post‐stroke (*n* = 8) and a control group (*n* = 8). Body mass at the time of muscle collection did not differ significantly between groups (post‐stroke: 465 ± 27 g vs. control: 495 ± 63 g). The study was approved by the Committee on Ethics of Animal Experimentation at the Vrije Universiteit Amsterdam (permit number: FBW 12‐01). After a series of contractile measurements described previously (Paudyal et al., 2021), the animals were euthanized with an overdose of intracardially‐injected pentobarbital sodium. The ECR and FCU muscles were quickly dissected from the affected limb and control animals, blotted dry, weighed, frozen—with vigorous shaking of the tissue until the nitrogen stopped bubbling—in liquid nitrogen and stored at −80°C until use.

### 2.2 Stroke induction

Photothrombotic stroke was induced as described previously (Paudyal et al., 2021). We assessed the preferred limb for grabbing a sugar pellet and then induced the stroke contralateral to the preferred limb (so the preferred limb was affected by the stroke).

To induce the stroke, rats were anesthetized through inhalation of isoflurane (induction 4%, maintenance 1.5%–2%) followed by a single dose of the painkiller buprenorphine (intraperitoneally; 0.01 mg kg^−1^ body mass; Temgesic; Schering–Plow, Maarssen, the Netherlands). The scalp was shaved, the head fixed in a stereotaxic frame in a prone position, and 2% lidocaine was injected subcutaneously at the incision site on the head. Under aseptic conditions, a midline incision of 2.0–2.5 cm was made through the scalp, and the skin was retracted laterally to expose the coronal and sagittal sutures. Once the bregma and lambda were exposed, the illumination area was defined as 1.5–4.5 mm lateral and + 4.0 to −4.0 mm anterior/posterior to the bregma. Two minutes after an intravenous injection of Rose Bengal (Sigma Aldrich) solution (25 mg kg^−1^ body mass) into the saphenous vein at a rate of 5.625 mL min^−1^ via a single syringe infusion pump (World Precision Instrument, SP100IZ), the halogen light with a 5‐mm diameter aperture (Schott KL 1500 LCD, Germany) with a green filter (wavelength 560 nm) was turned on for 20 min for transcranial illumination. The remaining exposed area of the skull was covered with black tape to prevent undesired illumination. After termination of isoflurane anesthesia, the condition of the rat was monitored for 2 h before they returned to their home cage. Body temperature was monitored continuously and maintained at 37°C by a heating pad. Our previous study showed that this procedure successfully resulted in an infarct of the target brain area that is, the forelimb region of the primary sensorimotor cortex, in all animals that impaired placement of the contralateral forelimb on a table when the contralateral vibrissae contacted the table (Paudyal et al., 2021). Using the skilled‐reach test (Moon et al., 2009; van Lieshout et al., 2021), we observed 3 days after stroke that the success rate of reaching a sugar pellet and placing it in the mouth at first attempt was reduced from 40% ± 14% to 7% ± 8% (*p* = 0.012, paired *t*‐test, *n* = 5), and similar to (van Lieshout et al., 2021) 40% of the animals were even unable to perform the task successfully. In addition, a study using the same model reported that impairments in skilled reaching performance were similar in this rat model and post‐stroke humans (van Lieshout et al., 2021).

### 2.3 Histochemistry

**FIGURE 1:**
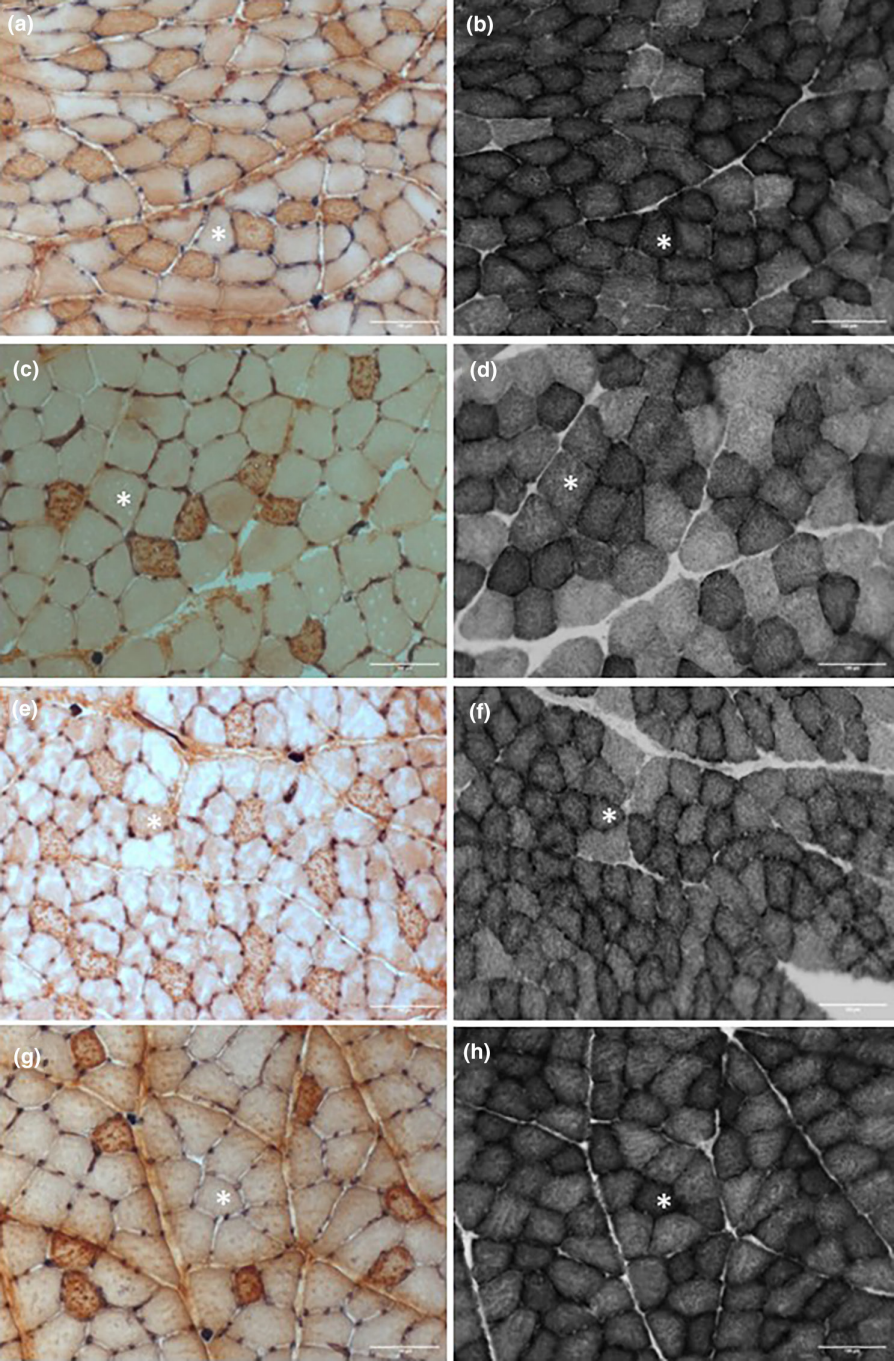
Histological pictures of serial sections from the *m. extensor carpi radialis* (ECR) (a, b, e, f) and *m. flexor carpi ulnaris* (FCU) (c, d, g, h) of control (a–d) and stroke (e–h) rats stained for capillaries and type I (dark) fibers (a, c, e, g), and succinate dehydrogenase (b, d, f, h). *Same fiber in serial section; scale bars 100 μm.

Serial 10‐μm cross‐sections of the muscles were cut in a cryostat at −20°C. Capillaries and type I fibers were co‐stained as described previously (Barnouin et al., 2017). Fibers not stained with the type I antibody were considered type II fibers. Briefly, after fixing the slides in ice‐cold acetone for 5 min and washing in HEPES buffer, the sections were blocked in 0.1% bovine serum albumin in HEPES for 60 min. After 15 min peroxide incubation, the sections were incubated with biotinylated *Griffonia simplicifolia* lectin (50 μg mL^−1^; Vector Laboratories, Peterborough, UK) in 1% bovine albumin‐HEPES with anti‐myosin heavy chain type I [0.41 μg∙mL^−1^, Developmental Studies Hybridoma Bank (DSHB, USA)] for 1 h to detect capillaries and type I fibers, respectively. Sections were then incubated with the secondary Vectastain anti‐mouse IgG antibody (Vector Laboratories, UK) and stained using the Vectastain ABC kit (Vector Laboratories). The sections were mounted in glycerol‐gelatine for further analysis. A serial section was stained for succinate dehydrogenase (SDH) as an estimate of oxidative capacity as described previously (Barnouin et al., 2017; Paudyal et al., 2018). Examples of the staining in the ECR and FCU of control and stroke rats are shown in Figure 1.

Both the ECR (Rodrigues Ade et al., 1994) and the FCU exhibit a more glycolytic superficial and a more oxidative deep region with more type I fibers, similar to that seen in the plantaris muscle (Degens et al., 1992). We therefore, in previous studies, analyzed the deep and superficial region separately (Degens et al., 1992; Hendrickse et al., 2020). This was not possible in the current study as previously (Paudyal et al., 2021) we assessed the number of sarcomeres in series in longitudinal sections of the FCU, leaving the superficial region for further analysis. That we indeed also selected the superficial region in the ECR is reflected by the percentage of type I fibers in the ECR that was never more than 27.4%. The region of interest (ROI) was on average 0.26 mm^2^ and contained 57–161 complete fibers, bar one section where due to technical problems only 42 fibers in a ROI of 0.14 mm^2^ were analyzed.

### 2.4 Capillarization

The method of capillary domains was used to analyze the capillarization in skeletal muscle (Barnouin et al., 2017; Paudyal et al., 2018). Capillary coordinates and fiber outlines were recorded with BTablet (Science Applications page L. Hoofd). The coordinates of capillaries and fiber outlines were then imported into AnaTis (Science Applications page L. Hoofd) to calculate capillary domains. A capillary domain is an area surrounding a capillary delineated by equidistant boundaries from surrounding capillaries (Hoofd et al., 1985) and is a good estimate of the capillary oxygen supply area (Al‐Shammari et al., 2014).

Using AnaTis, the fiber cross‐sectional area (FCSA), fiber shape (atrophied fibers may become more angular), capillary density (CD), and capillary‐to‐fiber ratio (C:F) were calculated. The shape of the fiber was given by the form factor, calculated as: perimeter^2^/(4π × FCSA), where higher values indicate a larger deviation from circularity. The variation in the FCSA was given as the standard deviation of the FCSA (SD FCSA). Fiber‐type proportion was expressed as the fiber number percentage (FNP). The standard deviation of log transformed domain areas (log_D_SD) was used as an index for heterogeneity of capillary spacing, which is a major factor for tissue oxygenation. The capillary domain method also allows one to calculate the capillary supply to individual fibers even when they lack direct capillary contact. The local capillary to fiber ratio (LCFR) of a fiber is given by the sum of domain fractions overlapping that fiber and considers that a capillary supplies more than one fiber. The capillary fiber density (CFD) of a fiber was calculated as the LCFR divided by the FCSA.

### 2.5 Oxidative capacity

The maximal oxygen consumption of a muscle fiber was determined as described previously (Barnouin et al., 2017; Bosutti et al., 2015; Des Tombe et al., 2002; Paudyal et al., 2018; van der Laarse et al., 1989). The optical density of SDH‐stained fibers at 660 nm (OD 660) was determined with ImageJ (ImageJ; NIH, USA). For each section, a separate calibration curve was constructed with a series of filters with a known optical density to prevent bias related to differences in background staining intensity, and lighting between sections and over time. The mass‐specific fiber maximal oxygen consumption (VO_2_max_mass‐specific_ in L kg^−1^ min^−1^) was calculated (assumption that 1 mol of oxygen is 22.4 L, and the density of muscle is 1 kg L^−1^) as:

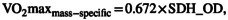

where SDH_OD is the optical density of the SDH stain. The VO_2_max per fiber was calculated as VO_2_max_mass‐specific_ × FCSA and gives the maximal oxygen consumption of a fiber in *p*L mm^−1^ min^−1^ (Bosutti et al., 2015). To assess the oxygen supply to demand ratio for each fiber type, we calculated the LCFR/(FCSA × SDH_OD).

### 2.6 Statistics

Data were analyzed with SPSS (Statistics version 22, IBM, Chicago, IL, USA). A mixed linear model was used as some data were missing on average data for each rat, with as within factors muscle and fiber type, and as between factor condition. Effects and interactions were considered significant at *p* < 0.05. All data are presented as mean ± SD.

## 3 RESULTS

### 3.1 Muscle mass and fiber characteristics

**FIGURE 2:**
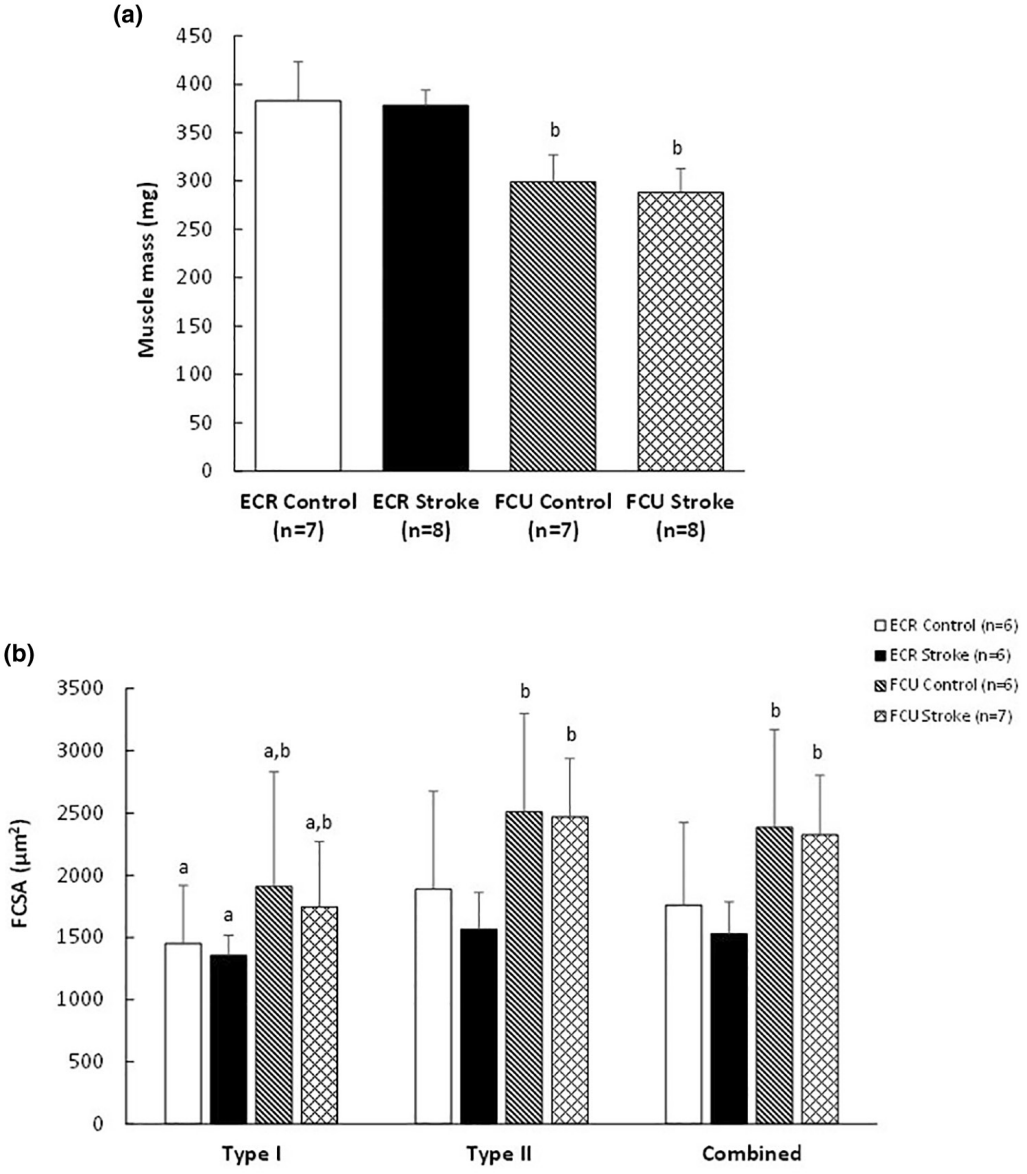
(a) Muscle mass (^b^differences between muscles at *p* < 0.001) and (b) fiber cross‐sectional area (FCSA) in the *m. extensor carpi radialis* (ECR) and *m. flexor carpi ulnaris* (FCU) of control and stroke rats. Values are mean ± SD; ^a^different from type II fibers, main effect *p* = 0.005; ^b^different from ECR, main effect *p* ≤ 0.001.

The ECR was heavier than the FCU (*p* < 0.001) but had smaller fibers (*p* = 0.001) (Figure 1). Type II fibers were larger than type I fibers (*p* = 0.005), but neither muscle showed a significant effect of stroke on mass or FCSA (Figure 2).

**FIGURE 3:**
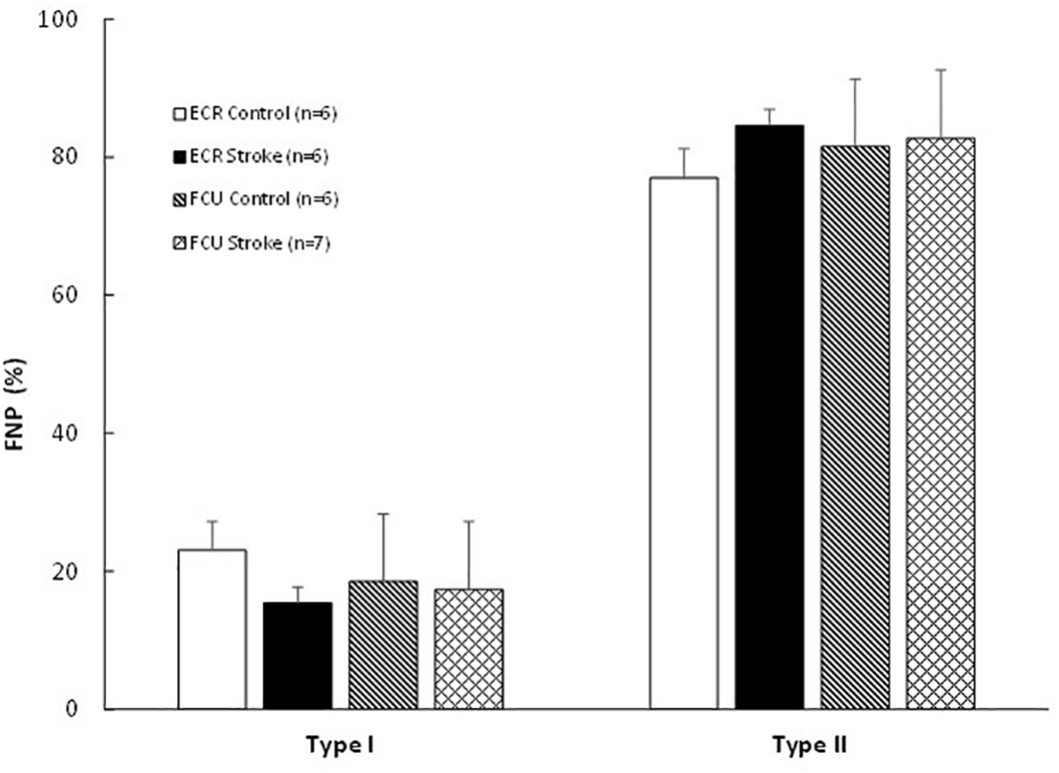
Fiber number percentage (FNP) (%) in the *m. extensor carpi radialis* (ECR) and *m. flexor carpi ulnaris* (FCU) of control and stroke rats. Values are mean ± SD.

The fiber type composition did not differ significantly between the FCU and ECR, and was not significantly affected by stroke in either muscle (Figure 3). The variation in fiber sizes, expressed as SD FCSA, was larger in the FCU than the ECR (*p* < 0.001) and in type II than type I fibers (*p* < 0.001) (Table 1). Neither the SD FCSA nor the shape factor were significantly affected by stroke (Table 1).

**TABLE 1:** Fiber size variation (SD FCSA) and shape factor in rat control *m. extensor carpi radialis* (ECR) and *m. flexor carpi ulnaris* (FCU) and after 4 weeks stroke.

	ECR		FCU		Effects (*p*‐values)			Interaction (*p*‐values)		
	Control (*n* = 6)	Stroke (*n* = 6)	Control (*n* = 6)	Stroke (*n* = 7)	Condition	Muscle	Type	CM	CT	MT
SD FCSA I	249 ± 82	200 ± 70	327 ± 189	279 ± 133	0.864	<0.001	<0.001	0.598	0.225	0.102
SD FCSA II	310 ± 70	310 ± 78	468 ± 113	541 ± 178						
Shape factor I	1.28 ± 0.06	1.32 ± 0.06	1.35 ± 0.17	1.27 ± 0.05	0.552	0.894	0.427	0.586	0.946	0.044
Shape factor II	1.32 ± 0.03	1.34 ± 0.05	1.35 ± 0.15	1.28 ± 0.05						

*Note*: Values are presented as mean ± SD.

Abbreviations: CM, condition × muscle type; CT, condition × fiber type; MT, muscle type × fiber type; FCSA, fiber cross‐sectional area.

### 3.2 Capillarization

**FIGURE 4:**
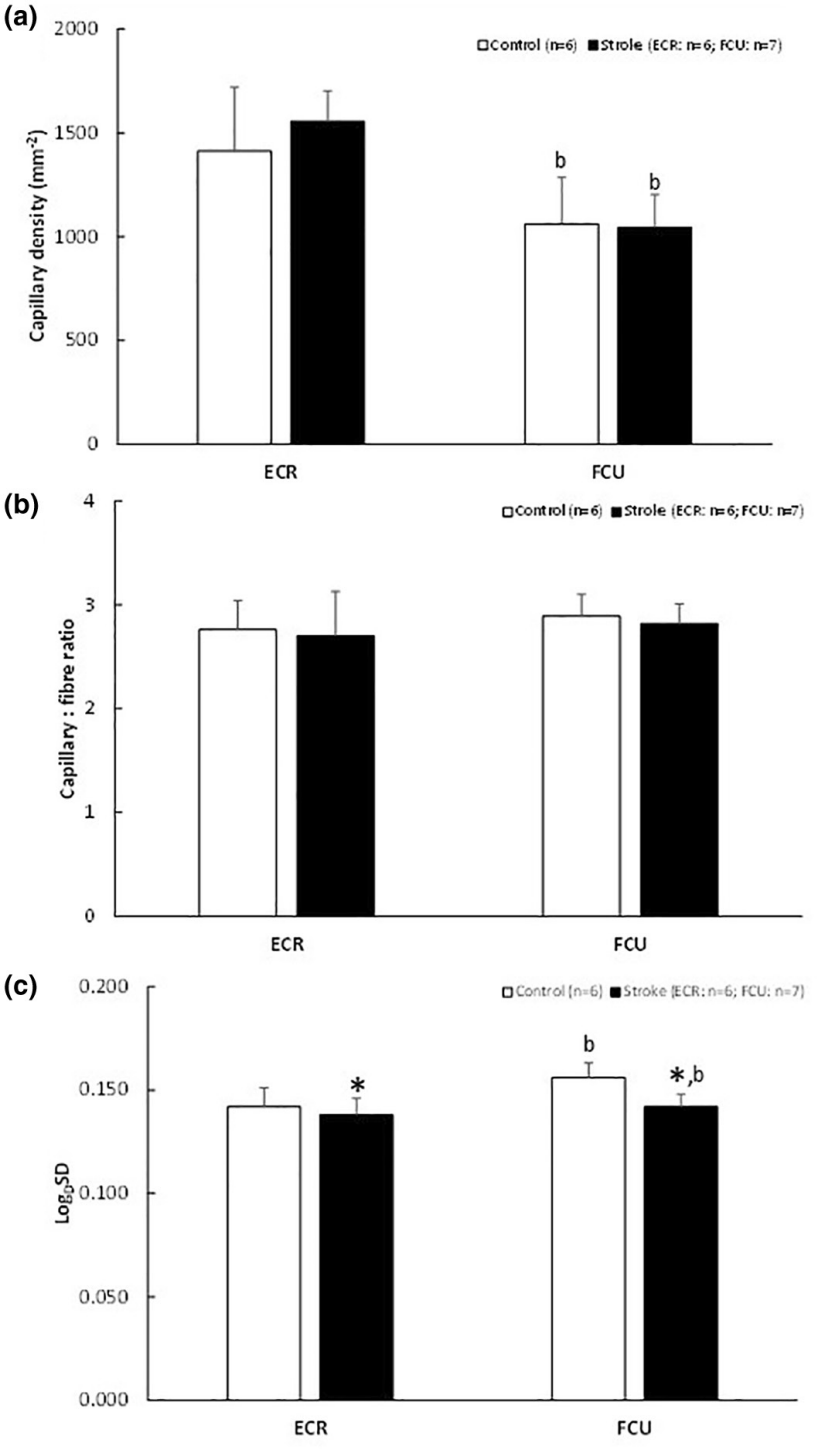
(a) Capillary density, (b) capillary‐to‐fiber ratio and (c) heterogeneity of capillary spacing (Log_D_SD) in *m. extensor carpi radialis* (ECR) and *m. flexor carpi ulnaris* (FCU) in control and stroke rats. Values are mean ± SD; ^b^different from ECR, main effect *p* < 0.01; *main effect of stroke at *p* = 0.006.

The CD, C:F, and heterogeneity of capillary spacing (Log_D_SD) are presented in Figure 4. The ECR had a higher CD than the FCU (*p* < 0.001), but there was no significant effect of stroke (Figure 4a). There was no significant difference in the C:F between the ECR and FCU, or between stroke and control muscles (Figure 4b). The Log_D_SD was higher in the FCU than the ECR (*p* = 0.006). There was a main effect of stroke on the Log_D_SD (*p* = 0.006) without a significant muscle‐stroke interaction (*p* = 0.144) indicating that the effect of stroke on the Log_D_SD was similar in the ECR and FCU: Log_D_SD was 2.8% and 9.0% lower in the ECR and FCU, respectively (*p* = 0.006; Figure 4c), indicating a more homogeneous distribution of capillaries after stroke.

**FIGURE 5:**
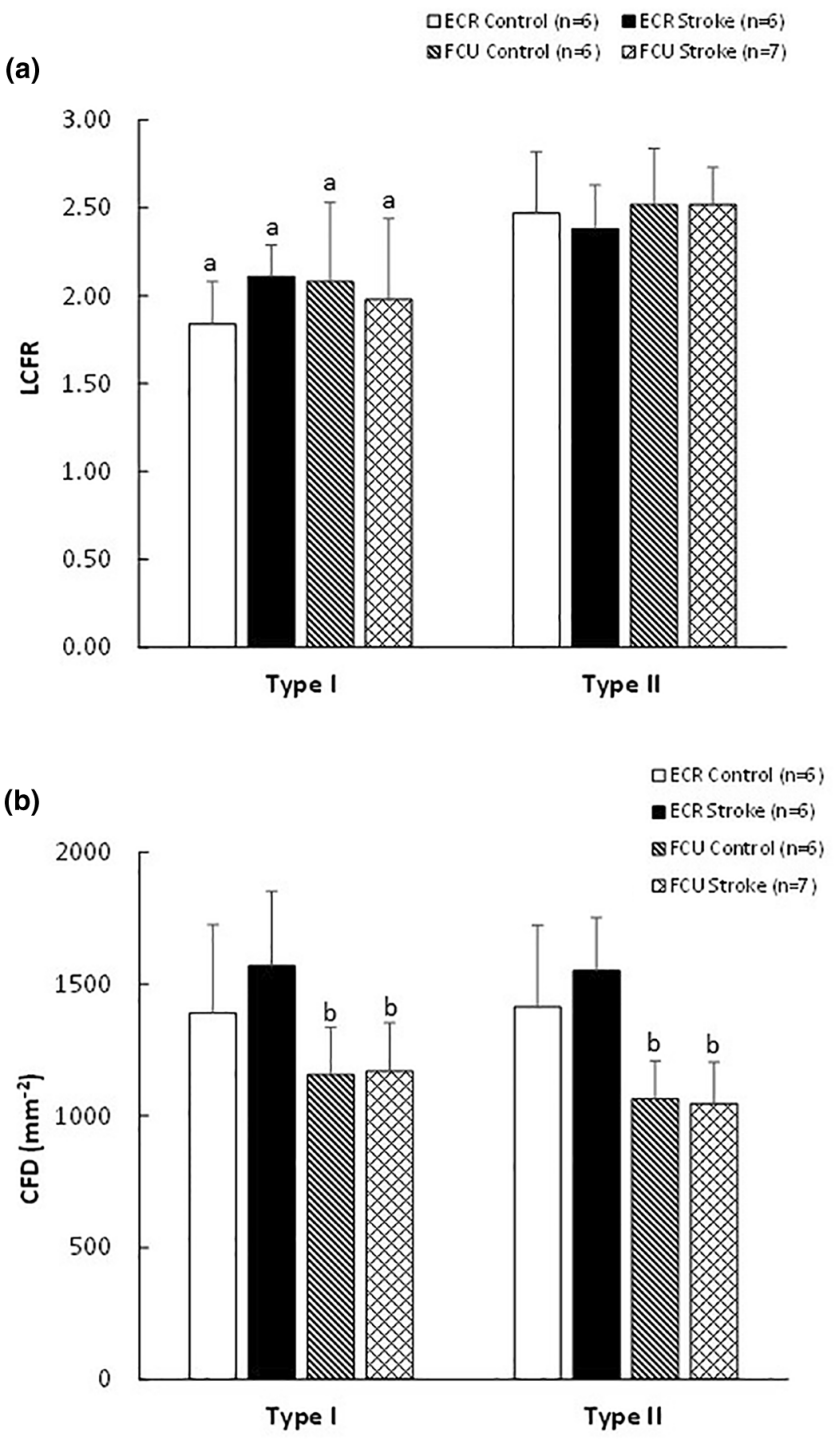
(a) Local capillary to fiber ratio (LCFR) and (b) capillary fiber density (CFD) in control and stroke *m. extensor carpi radialis* (ECR) and *m. flexor carpi ulnaris* (FCU). ^a^Different from type II fibers, main effect *p* < 0.001; ^b^different from ECR, main effect *p* < 0.001.

The LCFR was similar in the ECR and FCU, but type II fibers had a higher LCFR than type I fibers (*p* < 0.001; Figure 5a). The CFD was similar in type I and type II fibers, but was higher in the ECR than FCU (*p* < 0.001; Figure 5b). The LCFR (Figure 5a) and CFD (Figure 5b) were not significantly affected by stroke.

### 3.3 Oxidative capacity

**FIGURE 6:**
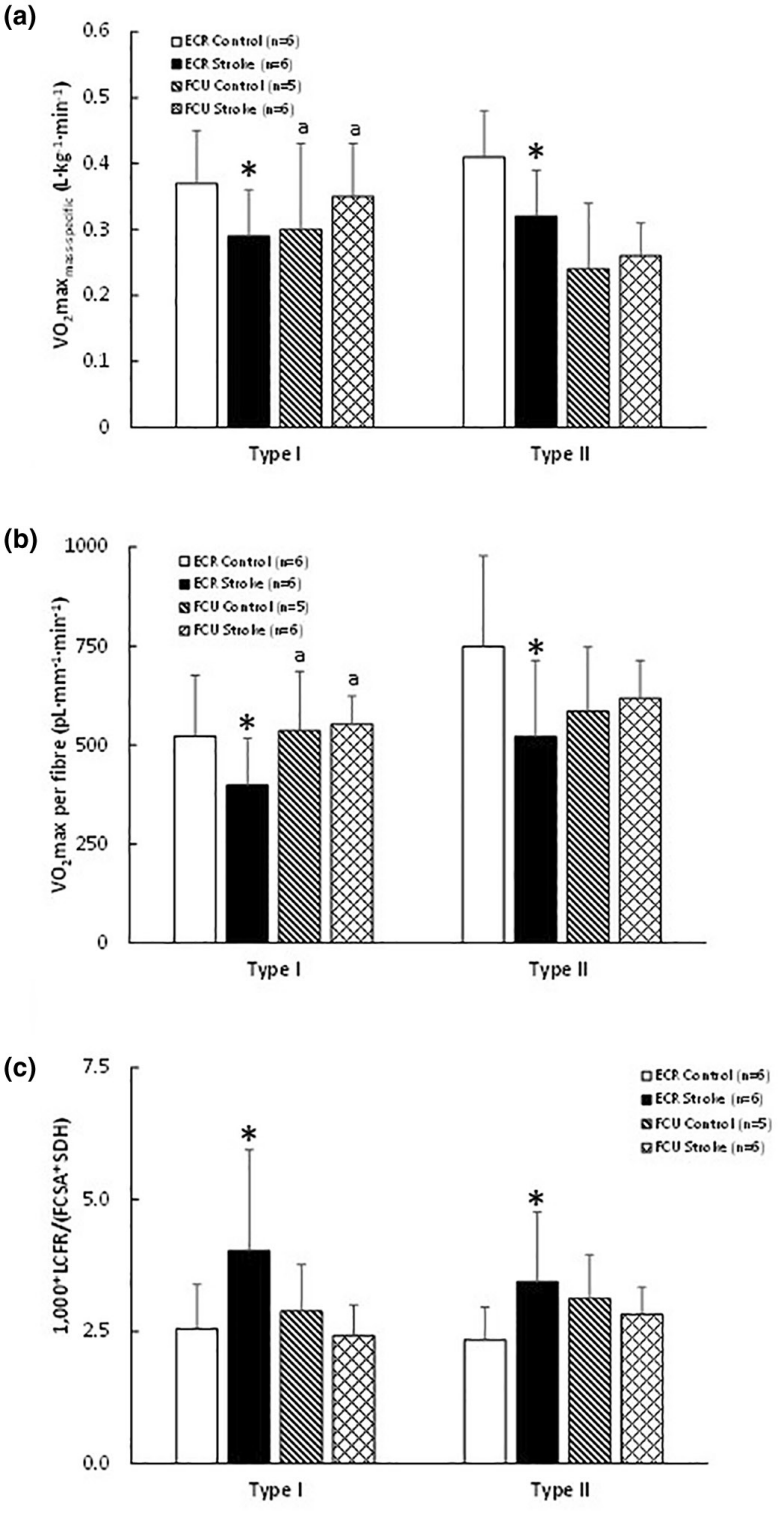
(a) Mass specific maximal oxygen consumption (VO_2_max_mass‐specific_), (b) maximal oxygen consumption of a fiber (VO_2_max per fiber), and (c) capillary‐to‐fiber ratio per optical density of succinate dehydrogenase stain and cross‐sectional area of the fibers (LCFR/(SDH × FCSA) in the *m. extensor carpi radialis* (ECR) and *m. flexor carpi ulnaris* (FCU) from control and stroke rats. ^a^Different from type II fibers in FCU only, main effect *p* = 0.022; *effect of stroke in ECR only at *p* < 0.03.

The condition × muscle interaction (*p* = 0.021) for VO_2_max_mass‐specific_ was reflected by a significant stroke‐related decrease in VO_2_max_mass‐specific_ in the ECR, but no significant effect of stroke in the FCU (Figure 6a). The VO_2_max per fiber for type II fibers was higher than that for type I fibers (*p* = 0.014). There was a significant condition × muscle interaction (*p* = 0.033), reflected by a stroke‐induced reduction in the ECR, but not in the FCU (Figure 6b).

There were no significant differences in LCFR/(FCSA × SDH) between fiber types (Figure 6c). However, there was a condition × muscle interaction (*p* = 0.01), reflected by an increase in LCFR/(FCSA × SDH) in ECR post‐stroke, but no significant change in the FCU (Figure 6c).

## 4 DISCUSSION

The main observations of the present study are that 4 weeks after stroke in rat there were (i) no significant changes in the cross‐sectional area and shape of the muscle fibers, and (ii) although there was no significant capillary rarefaction within the first 4 weeks after stroke, there was some evidence for remodeling of the capillary bed as reflected by a reduced heterogeneity of capillary spacing that may result in improved muscle oxygenation. (iii) In the ECR, but not in the FCU, this was accompanied by a reduction in muscle fiber oxidative capacity that resulted in an excess capillary supply in the ECR. This suggests that at least the early stroke‐induced adaptations are muscle specific.

### 4.1 Rodent stroke models

We induced stroke in the forelimb region of the primary sensorimotor cortex using a photothrombotic procedure (Paudyal et al., 2021). This is less invasive than the occlusion of the middle cerebral artery (Balch et al., 2021) and the pial strip method (Dormer et al., 2009). Like the pial strip method (Dormer et al., 2009), a major advantage of the photothrombotic stroke is that the location and size of the affected brain area can be controlled by the direction and size of the light beam. Indeed, a comparison with the literature suggests that middle cerebral artery occlusion leads to a more severe ischemic stroke than the photothrombotic stroke we applied (e.g., comparing Figure 2 in Paudyal et al., 2021 with Figure 1 in Balch et al., 2021 and Tuntevski et al., 2020) and hence allows a more precise assessment of impairments due to stroke in a specific area. It has been shown that the photothrombotic stroke in the forelimb region of the primary sensorimotor cortex induced impairments in skilled reach performance like those seen post‐stroke in humans (van Lieshout et al., 2021).

### 4.2 Fiber type composition and fiber size

Neither in the FCU nor in the ECR there were significant muscle fiber atrophy, changes in fiber type composition, increased variation in fiber sizes, nor an increased presence of angular fibers in the first 4 weeks post stroke. This is in contrast to the presence of angular fibers (Slager et al., 1985) and muscle atrophy often seen after stroke in humans (Adkins et al., 2021; Hunnicutt & Gregory, 2017; Ryan et al., 2002; Severinsen et al., 2016; Slager et al., 1985) and rodents (Choe et al., 2006; Springer et al., 2014; Tuntevski et al., 2020), and the shift from type I to type II fibers in rodent (Balch et al., 2021; Choe et al., 2006) and human (Prior et al., 2009; Qi et al., 2024; Severinsen et al., 2016) muscles. Others, however, did not observe significant muscle or muscle fiber atrophy (Prior et al., 2009; Sunnerhagen et al., 1999), or a fiber type shift in human (Sunnerhagen et al., 1999) or rodent muscles after stroke (Dormer et al., 2009; Paudyal et al., 2021).

It is unlikely that the time since stroke plays a role as other rodent studies have seen atrophy or a loss of force generating capacity within 1 week (Choe et al., 2006; Springer et al., 2014; Tuntevski et al., 2020) or 3 weeks after stroke (Balch et al., 2021), well before the 4 weeks in our study. Even in humans, muscle mass and strength were lower after stroke than in a reference population, but did not decline significantly between 3 days and 6 months post stroke (Carin‐Levy et al., 2006), and histological changes were independent on duration since stroke (9 months–25 years) (Slager et al., 1985), indicating that any changes, if present, occur early as has also been seen following bed rest (Hendrickse et al., 2022).

As it has been shown that cerebral infarct severity correlated with the catabolic activity in the affected leg (Springer et al., 2014), a likely explanation for the absence of significant changes in fiber type composition and fiber sizes is that middle cerebral artery occlusion leads to a more severe ischemic stroke than the photothrombotic stroke we applied. In addition to a direct effect of stroke severity, it is possible that a milder stroke does not cause a major decline in physical activity levels. Indeed, the stroke patients (Sunnerhagen et al., 1999) had a “relatively high level of physical activity” and in mice regular exercise attenuated the atrophy and the fiber type shift after acute middle cerebral artery occlusion (Choe et al., 2006). Whatever the cause of the absence of muscle atrophy and fiber type shifts, these data indicate that stroke is not necessarily accompanied with muscle wasting and shifts in fiber type composition, and that fiber atrophy and a type I to type II fiber type shift may be related to stroke severity and reductions in physical activity levels after stroke.

### 4.3 Oxidative capacity and capillarization

Both in human (Severinsen et al., 2016; Sunnerhagen et al., 1999) and in rodent muscle (Balch et al., 2021) a reduced muscle oxidative capacity has been observed after stroke, something we did see in the ECR but not in the FCU. A reduced oxidative capacity may well contribute to the attenuated desaturation during exercise (Hyngstrom et al., 2023). As the fatigue resistance of a muscle is related to muscle oxidative capacity (Degens & Veerkamp, 1994), such a reduction in oxidative capacity may underlie the reduced muscle fatigue resistance after stroke (Gerrits et al., 2009; Gianuzzi et al., 2001; Qi et al., 2024) and explain why in patients with a minor motor impairment there was neither a reduction in muscle oxidative capacity nor a reduced muscle fatigue resistance (Sunnerhagen et al., 1999). Perhaps the absence of a reduction in oxidative capacity in the FCU but not in the ECR is therefore related to different activity patterns, where the FCU is still being used to grab food while the ECR is recruited less after stroke. While it has been shown in rats that even low‐intensity exercise immediately after stroke is sufficient to attenuate significant fiber atrophy and the stroke‐related fiber type transition (Choe et al., 2006), it remains to be seen whether such exercise is also sufficient to minimize any loss of oxidative capacity.

In contrast to several human studies (Prior et al., 2009; Sunnerhagen et al., 1999), we did not observe a decline in CD. As only in the ECR, but not in the FCU, the fiber oxidative capacity was reduced after stroke, the capillary supply was in relative excess to the oxidative capacity in the ECR. An excess capillary supply, as a result of an earlier onset or faster reduction of oxidative capacity than capillary rarefaction, has also been seen in aging muscle (Hepple & Vogell, 2004), after bed rest (Hendrickse et al., 2022) and denervation (Paudyal et al., 2018). Such excess capillary supply has been shown to result in enhanced muscle oxygenation (Hendrickse et al., 2022) that will be further improved by the reduced heterogeneity of capillary spacing (Degens et al., 1994, 2006; Piiper & Scheid, 1991) we observed after stroke. An excess capillary supply may put the muscle in an advantageous position to develop hypertrophy and indeed older people with a lower CD had an attenuated hypertrophic response to resistance exercise (Snijders et al., 2017), an important consideration in rehabilitation after stroke.

In human muscle, there was a stroke‐induced reduction, rather than an increase, in CD (Prior et al., 2009; Sunnerhagen et al., 1999). This may be related to the duration since stroke, as at least resting blood flow may be transiently elevated, as seen for 21 days after denervation in rats (Eisenberg & Hood, 1994). Such a transiently increased flow will be associated with increased endothelial shear stress preventing capillary rarefaction (Hudlicka et al., 1992). However, in chronic stroke patients, a reduced resting (Ivey et al., 2010) and contraction‐induced blood flow (Murphy et al., 2019) has been reported. The consequent reduction in shear stress may contribute to capillary rarefaction resulting in a reduced CD that was associated with impaired glucose tolerance after stroke (Prior et al., 2009). It is therefore unlikely that the rats in our study suffered from glucose intolerance.

It has been suggested that capillary rarefaction in one (Prior et al., 2009) but not another study (Sunnerhagen et al., 1999) may be related to differences in motor impairment and hence perhaps stroke severity, where in the first study the average walking speed was 0.4 m/s while in the latter the speed was 1.6 m/s. Another possibility is therefore that, although it has been shown that our focal photothrombosis in rats and stroke in humans causes a reduced “skilled reaching performance,” this had recovered largely before 4 weeks (van Lieshout et al., 2021), and hence the transient reduction in motor impairment may have prevented significant capillary rarefaction.

### 4.4 Limitations

The sample size may have precluded the detection of significant differences. However, a power analysis revealed that we would have needed 46 animals per group to detect that the stroke‐induced muscle fiber atrophy was significant at a power of 0.80, indicating that the differences are very much within the normal range of fiber sizes. Our analyses were restricted to the superficial glycolytic region of the muscles with a preponderance of type II fibers. Although stroke‐induced adaptations may be different in the deep, more oxidative region of the muscle, we consider this unlikely as we did not see any stroke × fiber type interactions, suggesting that the effects observed are independent of fiber type. While the literature seems to indicate that muscle changes occur within the first 2 weeks after stroke, it will be worthwhile to assess the time course over longer periods after stroke.

### 4.5 Conclusion

A stroke that affects a limited area of the brain is not associated with significant muscle atrophy or capillary rarefaction, but does induce a muscle‐specific loss of oxidative capacity. Further studies are needed to reveal differences between stroke models and time points following stroke.

## FUNDING INFORMATION

This research was funded by the European Commission through MOVE‐AGE, an Erasmus Mundus Joint Doctorate programmme (2011‐0015).

## ETHICS STATEMENT

The study was approved by the Committee on Ethics of Animal Experimentation at the Vrije Universiteit Amsterdam (permit number: FBW 12‐01).

## Data Availability

The data that support the findings of this study are available from the corresponding author upon reasonable request.
